# Electroanatomical Conduction Characteristics of Pig Myocardial Tissue Derived from High-Density Mapping

**DOI:** 10.3390/jcm12175598

**Published:** 2023-08-28

**Authors:** Theresa Isabelle Wilhelm, Thorsten Lewalter, Johannes Fischer, Judith Reiser, Julia Werner, Christine Baumgartner, Lukas Gleirscher, Petra Hoppmann, Christian Kupatt, Klaus Tiemann, Clemens Jilek

**Affiliations:** 1 Peter-Osypka Heart Centre Munich, Internistisches Klinikum München Süd, 81379 Munich, Germanythorsten.lewalter@ikms.de (T.L.);; 2 Medical Graduate Center, School of Medicine, Technical University of Munich, 81675 Munich, Germany; 3 Center for Preclinical Research, University Hospital Rechts der Isar, Technical University of Munich, 81675 Munich, Germany; 4 Department of Internal Medicine I, University Hospital Rechts der Isar, Technical University of Munich, 81675 Munich, Germany

**Keywords:** conduction velocity, voltage, ultra-high-density mapping, heart, pig

## Abstract

Background: Ultra-high-density mapping systems allow more precise measurement of the heart chambers at corresponding conduction velocities (CVs) and voltage amplitudes (VAs). Our aim for this study was to define and compare a basic value set for unipolar CV and VA in all four heart chambers and their separate walls in healthy, juvenile porcine hearts using ultra-high-density mapping. Methods: We used the Rhythmia Mapping System to create electroanatomical maps of four pig hearts in sinus rhythm. CVs and VAs were calculated for chambers and wall segments with overlapping circular areas (radius of 5 mm). Results: We analysed 21 maps with a resolution of 1.4 points/mm^2^. CVs were highest in the left atrium (LA), followed by the left ventricle (LV), right ventricle (RV), and right atrium (RA). As for VA, LV was highest, followed by RV, LA, and RA. The left chambers had a higher overall CV and VA than the right. Within the chambers, CV varied more in the right than in the left chambers, and VA varied in the ventricles but not in the atria. There was a slightly positive correlation between CVs and VAs at velocity values of <1.5 m/s. Conclusions: In healthy porcine hearts, the left chambers showed higher VAs and CVs than the right. CV differs mainly within the right chambers and VA differs only within the ventricles. A slightly positive linear correlation was found between slow CVs and low VAs.

## 1. Introduction

Cardiac arrhythmias are a disruption of the normal cardiac rhythm and can range from simple changes in heart rate to complex fibrillation events. They may result in various clinical symptoms, from reduced physical resilience to sudden cardiac arrests [1]. Atrial fibrillation is the most common sustained arrhythmia, affecting 0.51% of the world’s population. Its worldwide prevalence has increased by 33% over the past 20 years and is expected to rise by >60% in the coming 30 years [2].

Re-entry mechanisms cause many cardiac arrhythmias. The zones of slow conduction play a key role in developing and maintaining reentrant tachycardias [1,3,4,5]. Therefore, treatment by catheter ablation involves the ablation of slow conduction or low voltage zones [6] that can be identified with ultra-high-density mapping systems [7].

Until recently, there have been few, mostly incomplete data on conduction velocities in healthy hearts.

Hansson et al. collected data from the right atrial wall with an epicardial electrode array [8]. Martin et al. measured conduction velocities and electrogram amplitudes in different sections of re-entry circuits, mainly among patients with ischemic cardiomyopathy [5]. Kléber et al. observed the time course of left ventricular conduction velocity changes during induced ischemia in isolated porcine hearts [9].

None of these studies provided an overall approach with reference values or comparisons between physiological conduction velocity and voltage amplitude in all four heart chambers. Characterizing normal heart electrical physiology is crucial to distinguish between physiological and diseased patterns.

In this study, we aimed to define and compare a basic value set for unipolar conduction velocities and voltage amplitudes in all four heart chambers and their separate walls in healthy, in vivo porcine hearts using ultra-high-density mapping. In addition, we analysed whether conduction velocity and voltage amplitude are correlated in healthy hearts. Our velocity calculations for the three-dimensional myocardial surface were based on overlapping circular areas.

## 2. Materials and Methods

Experimental data were collected from four juvenile healthy swine (German Landrace x Pietrain; 31–41 kg (mean 36 kg), 3–4 months old), two of which were female (pigs 3 and 4). The study was approved by the government of Bavaria (ROB-55.2-2532.Vet_02-1 7-174).

### 2.1. Electroanatomical Mapping

Electrophysiological studies were performed in vivo under general anaesthesia and mechanical ventilation during intrinsic sinus rhythm. Sedation was administered intramuscularly with ketamine (10–15 mg/kg), azaperone (2 mg/kg), and atropine (1 mg). Anaesthesia was introduced with 1% and maintenance with 2% propofol i.v. We administered acetylsalicylic acid (250 mg i.v.) and heparin (150 IE/kg i.v. as a bolus and 200 IU/mL i.v. as continuous drip infusion depending on activated clotting time) for intraoperative anticoagulation after the sheath was placed. Intraoperative analgesia was provided by fentanyl boluses (0.015 mg/kg i.v.) every 20–30 min, and metamizole (40–50 mg/kg i.v.) was administered before the first incision. Transvenous catheters were inserted under fluoroscopic guidance. Access to the left heart was gained via a transseptal approach. Electroanatomical mapping was performed during sinus rhythm using Rhythmia, an ultra-high-density mapping system (Boston Scientific Corp., Marlborough, MA, USA), and its proprietary, 64-lead, multi-electrode basket mapping catheter Intellamap Orion (Boston Scientific Corp., USA). The sedation dosage was constant throughout the whole period of mapping to ensure comparable conditions between the different maps. The catheter is bidirectionally steerable and consists of eight splines, each containing eight electrodes spaced 2.5 mm apart [10].

### 2.2. Post-Processing in Rhythmia

**Figure 1:**
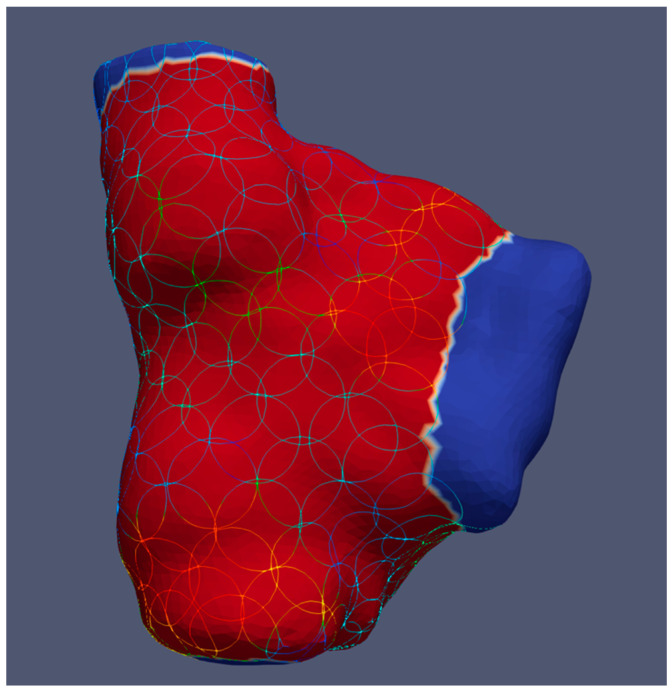
Example of a right atrium showing circular areas for calculating local conduction velocities and voltage amplitudes in ParaView. The blue colour indicates transitions to the venae cavae and right ventricle, where no circles are placed.

After completing the electrophysiological studies, the annotated beats were manually checked for plausibility and, if necessary, reannotated in Rhythmia to specifically ensure that no His–Purkinje-system signals were falsely annotated. Transitions to other cardiac chambers, arteries, and veins were identified based on the morphology and amplitude of electrograms and marked as cutouts (blue in Figure 1). The mean heart rate was calculated for each mapping procedure.

### 2.3. Calculation of Local Conduction Velocity and Voltage Amplitude

To compute local conduction velocities and voltage amplitudes from the measured activation times while considering the variability of wavefront directions, we defined circular areas with an approximately 5 mm radius on the map surfaces and left out the cutouts. We used spherical filters from ParaView (v.5.8.0 and 5.10.0, Kitware Inc., New York, NY, USA) to create circular areas on the curved heart surface [11,12], as shown in Figure 1. Additional simulations were created for each heart chamber, where the circles only cover the septal, lateral, posterior, or anterior region in the case of ventricles plus the superior of the atria, respectively.

The mean voltage amplitude and conduction velocity of the covered map surface were calculated for each sphere. We divided the theoretical circle diameter, determined from the actual circular surface area, by the time difference between the first and last activations in each area to calculate conduction velocity, as seen in (1).

(1) $$conduction\;velocity=\frac{2\times \sqrt{\frac{area}{\pi }}}{∆\;activation}\;$$

All values and metadata were calculated and exported from ParaView using a custom-written Python script.

### 2.4. Statistical Analysis

Statistical analysis was performed using the statistical programming language R, v4.1.2 [13].

The relationship between conduction velocity and voltage amplitude, respectively, with the two factors mapping location and heart rate, was investigated by means of linear mixed effects analysis using lme4 [14,15] since repeated measurements, and a varying number of observations were obtained from four individuals. Mapping location and heart rate were used as fixed effects (with no interaction term). As a random effect, we used random intercepts for the pigs to account for inter-individual variation. The velocity was log-transformed in the linear model to obtain a normal distribution.

We used the lmerTest [16,17] and emmeans [18] packages for post hoc tests and comparisons, i.e., to compare the estimated mean values of the response variables for all levels of the explanatory variable under consideration. Tukey’s HSD was used for all pairwise comparisons and Sidak for targeted comparisons to adjust for multiple comparisons.

Post hoc test results are reported on the original scale. All estimated values are provided for a heart rate of 90 bpm. Estimates are reported in the format (estimate ± standard error, *p*-value). Correlations were tested via Pearson’s correlation coefficient, reported in the format (r (degrees of freedom) = r-statistic, *p* = *p*-value). Velocity values of >6 m/s were defined as outliers and ignored in the calculations. The *p*-values of <0.05 were considered significant. Measured amplitude values are reported as unipolar signals.

## 3. Results

A total of 21 maps were analysed, each consisting of 5632 ± 295 measurement points. The map surfaces had 131 ± 8 spheres, each covering an area of 77.8 mm^2^ ± 0.05, including 105 ± 0.19 measurement points. Therefore, the mapping resolution was 1.4 points/mm^2^. The volumes in Table 1 correspond to three-dimensional electroanatomic maps. Since these measurements also include transitions to the other chambers and adjacent parts of the vessels (excluded in the further calculations), the calculated volumes overestimate the actual size of the chambers, especially for the right atria.

### 3.1. No Influence of Sex on Conduction Velocity and Voltage Amplitude

Since the study was performed on two male and two female pigs, we performed a linear mixed-effects analysis to test whether sex affected conduction velocity and voltage amplitude. In our study, sex had no influence on conduction velocity (*p* = 0.50) or voltage amplitude (*p* = 0.30).

### 3.2. Mean Velocity and Voltage of Heart Chambers during Sinus Rhythm

The overall unipolar conduction velocity at a heart rate of 90 bpm is highest in the left atrium (LA) (0.79 ± 0.05 m/s), followed by the left ventricle (LV) (0.59 ± 0.04 m/s), the right ventricle (RV) (0.54 ± 0.03 m/s), and the right atrium (RA) (0.50 ± 0.03 m/s).

**Figure 2:**
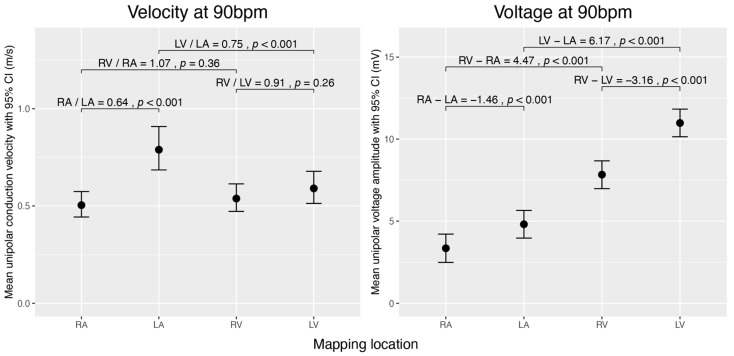
Comparison of estimated unipolar conduction velocities and voltage amplitudes during sinus rhythm across the cardiac chambers (RA, LA, RV, and LV) normalized to 90 bpm. The left graph displays mean conduction velocities (m/s), and the right graph shows mean voltage amplitudes (mV) with 95% confidence intervals. Brackets denote selected pairwise comparisons between chambers, with *p*-values testing if the ratio of mean velocities equals 1 or the difference in mean voltages equals 0 mV. The data were obtained using linear mixed-effects models and analysed using estimated marginal means. For velocity, we analysed the data on the log scale and then back-transformed the results for interpretation.

The overall unipolar voltage amplitude at a heart rate of 90 bpm is highest in the LV (10.98 ± 0.34 mV), followed by the RV (7.83 ± 0.32 mV), LA (4.81 ± 0.35 mV), and RA (3.35 ± 0.31 mV) (Figure 2).

### 3.3. Velocity and Voltage Differences between Chambers

#### 3.3.1. Inter-Atrial and Inter-Ventricular Comparison

We compared conduction velocities and voltage amplitudes between the atria and between the ventricles. The RA conducts significantly slower (×0.64 ± 0.03, *p* < 0.001) and has a lower voltage amplitude (−1.46 mV ± 0.19, *p* < 0.001) than the LA. By contrast, the voltage amplitude is higher in the LV than in the RV (−3.16 mV ± 0.21, *p* < 0.001), and no significant difference in conduction velocity was observed between the ventricles (Figure 2).

#### 3.3.2. Comparison between Atria and Ventricles of the Left and Right Heart

We then compared the conduction velocity and voltage amplitude between the atrium and ventricle of the same half of the heart. In the left chamber of the heart, the conduction velocity was higher in the atrium than in the ventricle (×0.748 ± 0.041, *p* < 0.001). We found no significant difference in conduction velocity between RA and RV. Voltage amplitudes were generally lower in the atria than in the ventricles (*p* < 0.001) (Figure 2).

### 3.4. Regional Velocity and Voltage Characteristics within Each Chamber

Each heart chamber was divided into subregions representing different walls. The following conduction velocity and voltage amplitude estimates are normalized to a heart rate of 90 bpm.

#### 3.4.1. Conduction Velocity

In the RA, the conduction velocity was highest in the posterior wall (0.61 ± 0.04 m/s), compared to the superior (0.42 ± 0.04 m/s, *p* < 0.044), lateral (0.44 ± 0.03 m/s, *p* < 0.001), and septal walls (0.48 ± 0.03 m/s, *p* = 0.009). In the RV, the anterior wall (0.44 ± 0.04 m/s) showed the lowest conduction velocity and was significantly different from the posterior (0.83 ± 0.08 m/s, *p* < 0.001), lateral (0.69 ± 0.07 m/s, *p* < 0.001), and septal walls (0.63 ± 0.06 m/s, *p* = 0.008). All other walls within these right heart chambers did not differ significantly.

**Figure 3:**
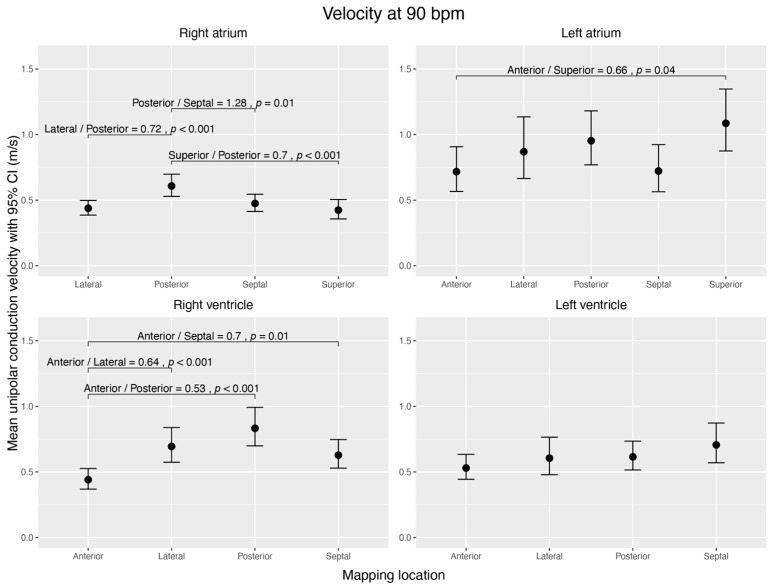
Visualization of estimated mean unipolar conduction velocities (m/s) during sinus rhythm, normalized to 90 bpm, across distinct regions of each cardiac chamber and their pairwise comparisons. The figure shows four subplots, each representing a cardiac chamber (right atrium, left atrium, right ventricle, and left ventricle), with potential mapping regions (anterior, posterior, lateral, septal, and superior). The 95% confidence interval is given for each mean velocity. Brackets highlight significant contrast ratios (*p* < 0.05) between the regions within the corresponding chamber, indicating divergence from a ratio of 1. The data were obtained using linear mixed-effects models and estimated marginal means. We conducted tests on the log scale and subsequently back-transformed the results for interpretation.

Within the LA, the superior wall had the highest conduction velocity (1.09 ± 0.12 m/s) and was significantly faster than the anterior wall, which had the slowest conduction velocity (0.72 ± 0.09, *p* = 0.043). We did not find any significant difference in conduction velocity between any other regions of the LA and LV (Figure 3).

#### 3.4.2. Voltage Amplitude

The atria showed no voltage differences between different walls.

The RV had high voltage amplitudes at the posterior (9.59 ± 0.52 mV) and septal walls (10.64 ± 0.52 mV), which were significantly higher than at the anterior (5.95 ± 0.52 mV) and lateral (6.73 ± 0.54 mV) walls (*p* < 0.001).

**Figure 4:**
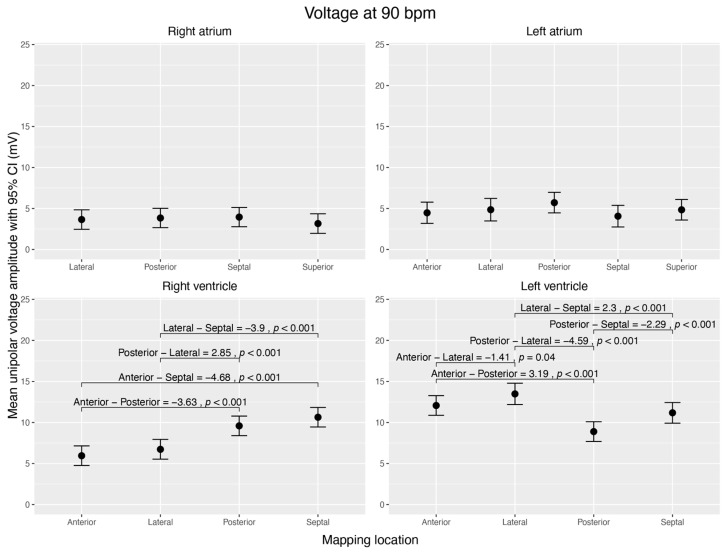
Visualization of estimated mean unipolar voltage amplitudes (mV) during sinus rhythm, normalized to 90 bpm, across distinct regions of each cardiac chamber and their pairwise comparisons. The figure shows four subplots, each representing a cardiac chamber (right atrium, left atrium, right ventricle, and left ventricle), with potential mapping regions (anterior, posterior, lateral, septal, and superior). The 95% confidence interval is given for each mean voltage. Brackets highlight significant voltage mean differences (Δ > 0 mV; *p* < 0.05) between the regions within the corresponding chamber. Data were derived from linear mixed-effects models with estimated marginal means.

Within the LV, the posterior wall (8.89 ± 0.52 mV) had the lowest voltage amplitude and was significantly different from any other (*p* < 0.001). The lateral wall (13.48 ± 0.61 mV) had the highest voltage amplitude (anterior vs. lateral *p* = 0.041; posterior vs. lateral *p* < 0.001; septal vs. lateral *p* < 0.001) (Figure 4).

### 3.5. Correlation of Velocity and Voltage

No general linear correlation between voltage amplitude and conduction velocity (r(2736) = 0.05, *p* = 0.008) was observed (|r| < 0.2).

**Figure 5:**
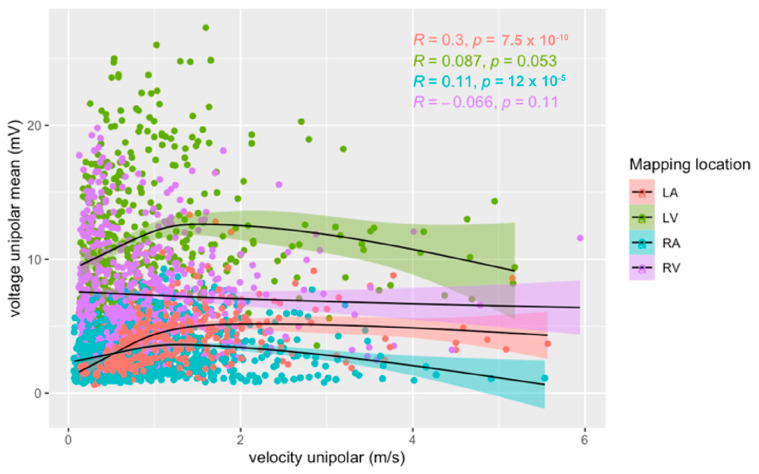
Relationship between unipolar voltage amplitude and unipolar conduction velocity for right (blue) and left (red) atria, and right (purple) and left (green) ventricles during sinus rhythm. Pearson’s correlation coefficients and their *p*-values for each heart chamber are provided.

Separated by mapping location, we found a low positive linear correlation between voltage amplitude and conduction velocity (r(408) = 0.30, *p* < 0.001) for the LA. In the other heart chambers, no linear correlation was observed (RA r(1252) = 0.11, *p* < 0.001; LV r(496) = 0.09, *p* = 0.053; RV r(574) = −0.07, *p* = 0.114) (Figure 5).

At velocities of <1.5 m/s, a low positive linear correlation between voltage amplitude and conduction velocity was observed in the LA (r(310) = 0.47, *p* < 0.001), LV (r(424) = 0.23, *p* < 0.001), and RA (r(1105) = 0.25, *p* < 0.001). No correlation was found in the RV (r(496) = −0.09, *p* = 0.05).

## 4. Discussion

To our knowledge, the current study is the first to use an ultra-high-density mapping system to systematically analyse intrinsic conduction velocities and voltage amplitudes in healthy pig hearts.

Our first key finding was that the highest conduction velocity of the heart chambers was found in the LA, whereas the highest amplitudes were measured in the LV. The left heart chambers showed higher voltage amplitudes and conduction velocities than their right counterparts. One explanation might be that a high conduction velocity in the left heart chambers is crucial to ensuring a synchronized electrical excitation of the chambers because of their larger size and greater myocardial mass [19]. As gap junctions seem to be the main determinant of conduction velocity in healthy myocardium [1], our observation may lead to the hypothesis that gap junction coupling might be higher in left-sided myocardium than right-sided myocardium.

Our second key finding was that within one heart chamber, conduction velocity varied more in the right heart chamber than in the left. In the RA, the posterior wall showed higher conduction velocities than the septal, lateral, and superior wall segments. In the RV, the anterior wall showed lower conduction velocities than the lateral, posterior, and septal wall segments. Within the LA, only the superior and anterior walls differed significantly, with the superior wall conducting the fastest. We interpret this finding as a measurement of the fast-conducting Bachmann bundle, which confirms the validity of our data [20,21]. The LV did not show any significant differences in conduction velocities between different segments.

Our third key finding was that there was no significant difference in voltage amplitude within the atria, except for one within the ventricles. In the RV, the posterior and septal walls showed higher voltage amplitudes than the anterior and lateral walls. In the LV, the lateral wall showed the highest voltage amplitude, whereas the posterior wall showed the lowest. The amplitudes in all segments of the ventricles were in the high range of >5 mV. In other words, no relevant low-voltage areas could be identified in absolute numbers. Further investigations should reveal whether differences in this high-amplitude range correspond to physiological structures.

Our fourth key finding was that in healthy, juvenile pig hearts, there was a slightly positive correlation between conduction velocity and voltage amplitude at velocities <1.5 m/s in all chambers except the RV. This finding might be due to the correlation between slow conduction zones and low-voltage areas in partially scarred myocardium [22,23].

The mapping resolution was very high compared to the published data. On average, the maps in our analysis showed a mapping resolution of 1.4 points/mm^2^ compared to the resolutions of 0.2 points/mm^2^ in another work characterizing left atrial slow conduction zones among patients with atrial fibrillation [24]. The number of mapping points is crucial to identifying critical ablation targets [7].

Our data are comparable to published human data on conduction velocities derived from electrodes with small surfaces and small inter-electrode distances. Martin et al. measured the conduction velocities among patients with ischemic cardiomyopathy [5]. Kléber et al. observed that myocardial tissue showed an ischemia-induced slowing of conduction velocity [9]. In contrast, Sanders et al. compared a prolonged conduction velocity in the right lateral atrium of patients with sinus node diseases to a matching group of patients with normal sinus node function. They conducted a second comparative analysis among patients with congestive heart failure and another matching group. The conduction velocities were 10 times higher than the pigs’ velocities in our study. The reason for this may lie in the mapping technique used by Sanders at the time, which was a non-high-density 3D mapping system [25,26] with a very rough analysis of conduction velocity.

In our analysis, we used unipolar signals, not bipolar signals, to calculate ultra-high-density 3D voltage maps. Moreover, unipolar mapping has been useful for identifying alterations in voltage amplitude, reflecting histologically proven viable myocardium in the scar areas of ischemic origin [27], as well as low-voltage areas in the LA.

### Limitations

Our data are derived from healthy porcine hearts, and their validity might be limited when transferred to human hearts. Healthy human heart measurements and outside interventions for all four heart chambers remain scarce for ethical reasons. Studies describing models to estimate local atrial conduction velocities [28] may develop and verify their models with the help of porcine heart data. Since pigs’ cardiovascular system, heart size, and body weight are similar to those of humans, we used a pig model as a valuable preclinical model [29]. The volumes in our study are smaller than in adult human hearts (RA 100 mL [30], LA 129 ± 44 mL [31], RV 44–101 mL [32], LV 58–120 mL [33]), possibly due to the juvenile age of the pigs (31–41 kg, 3–4 months old).

The porcine hearts were healthy and juvenile. Correlations between conduction velocities and voltage amplitudes that are presumed and observed in diseased hearts may be absent in healthy hearts. Therefore, conclusions regarding diseased and aged hearts can be misleading.

The sample size was limited to four pigs due to animal welfare. Despite using the random effect ‘heart’, we cannot rule out the possibility that strong inter-individual differences interfered with our statistical model.

The signals were recorded with one ultra-high-density mapping system and one distinct mapping catheter. There are several mapping systems on the market with different mapping catheter designs. As there are no head-to-head comparisons between different ultra-high-density mapping systems, limitations or advantages are unknown with regard to signal quality.

Since the velocity was calculated using time differences between the first and last excitations in the circle, colliding wavefronts might be misinterpreted. The probability of colliding wavefronts in healthy hearts should be low. The small radius of the circles prevents disturbances in analysing velocities due to colliding wavefronts.

The heart chambers and different segments of each heart chamber were mapped consecutively. Therefore, an overtime effect on velocities or voltages cannot be fully excluded. We tried to keep the influence of time low by adhering to strict timelines for mapping. Furthermore, we did not administer any anti-arrhythmic drugs that could influence cardiac electrophysiology.

Heart rate was estimated over a recording period of approximately 5 min, which may cause inaccuracies in the heart rate analysis. To allow a better interpretation of results, we assumed a linear relation in our mixed-effects model, which provided a good approximation of higher-dimensional interactions.

Anaesthesia has been shown to influence cardiac electrophysiology [34,35,36,37]. The pigs were mapped under stable sedation to ensure comparability with the electrophysiological baseline circumstances. Specifically, we cannot rule out systematic bias in the absolute values of conduction velocity.

## 5. Conclusions

In healthy porcine hearts, conduction velocities and voltage amplitudes differ between the left and right heart chambers. Within each heart chamber, voltage amplitude differs only in the ventricles and conduction velocity differs more in the right than in the left chambers.

There is a slightly positive correlation between conduction velocity and voltage amplitude at velocities of <1.5 m/s in the atria and LV of healthy porcine hearts.

A comprehensive characterization of the conduction velocities and voltage amplitudes of all heart chambers could facilitate future computations for human heart models.

## Figures and Tables

**Table 1:** Overview of the recorded maps, the mean heart rate during the procedure, the number of recorded measurement points, map volume, and the number of circles in whole maps and regions of each map.

Pig	Map-Nr.	Mapping Location	Heart Rate	Nr. of Points	Volume (mL)	Nr. of Circles Whole Map	Lateral Circles	Septal Circles	Posterior Circles	Anterior Circles	Superior Circles
1	1	RA	115	6839	77.65	184	30	23	26	-	9
1	4	RA	115	6907	77.65	184	30	23	26	-	9
1	5	LV	122	4806	53.16	122	10	14	20	21	-
1	9	LV	130	4726	53.16	122	10	14	20	21	-
2	10	RA	120	7475	69.66	167	21	17	16	-	11
2	12	RA	134	7475	69.66	167	21	17	16	-	11
2	14	LV	120	5060	50.10	129	11	13	26	24	-
2	16	LV	86	5060	50.10	129	11	13	26	24	-
2	18	LA	86	4835	39.95	100	11	10	16	12	15
2	20	LA	90	4964	40.68	100	11	10	16	12	15
2	21	RV	104	6440	68.88	138	12	16	17	21	-
2	23	RV	95	6445	68.64	138	12	16	17	21	-
3	25	RA	130	7815	81.00	193	30	35	33	-	10
3	26	RV	135	3547	33.95	74	9	13	9	14	-
3	32	RV	150	4698	35.82	66	10	9	6	6	-
5	36	RA	95	6848	64.54	176	41	21	21	-	13
5	37	RV	110	3158	25.93	84	12	15	16	9	-
5	39	RA	145	6799	69.00	186	45	32	26	-	24
5	42	RV	132	3420	28.04	84	12	15	16	9	-
5	44	LA	145	5481	40.70	105	4	9	10	9	11
5	45	LA	171	5481	40.70	105	4	9	10	9	11

## Data Availability

The data presented in this study are available on request from the corresponding author.
